# Contribution of the Bioball^TM^ head–neck adapter to the restoration of femoral offset in hip revision arthroplasty with retention of a well-fixed cup and stem

**DOI:** 10.1007/s00264-023-05833-7

**Published:** 2023-05-15

**Authors:** Clara Chimeno, Jenaro Ángel Fernández-Valencia, Alfonso Alías, Adrià Serra, Yury Postnikov, Andrés Combalia, Ernesto Muñoz-Mahamud

**Affiliations:** 1 Servei de Cirurgia Ortopèdica i Traumatologia, Hospital Clínic de Barcelona, Universitat de Barcelona (UB), C/ Villarroel 170, 08036 Barcelona, Spain; 2 Departament de Cirurgia i Especialitats Medicoquirúrgiques, Facultat de Medicina i Ciències de la Salut, Universitat de Barcelona (UB), C/ Casanova 143, 08036 Barcelona, Spain; 3 Facultat de Medicina i Ciències de la Salut, Universitat de Barcelona (UB), C/ Casanova 143, 08036 Barcelona, Spain; 4 Institut d’Investigacions Biomèdiques August Pi i Sunyer (IDIBAPS), C/ Villarroel 170, 08036 Barcelona, Spain

**Keywords:** Hip revision arthroplasty, Femoral offset, Bioball^TM^ head–neck adapter

## Abstract

**Purpose:**

Failure to restore the femoral offset of the native hip is a potential cause of dysfunctional hip arthroplasty. The aim of this study was to report our experience of using a modular head–neck adapter in revision THA, specifically analyzing its usefulness as a tool to correct a slightly diminished femoral offset.

**Materials and methods:**

This was a retrospective single-center study including all hip revisions performed at our institution from January 2017 to March 2022 where the BioBall^TM^ head–neck metal adapter was used. The preoperative and one year follow-up modified Merle d’Aubigné hip score was used to evaluate functional outcomes.

**Results:**

Of a total of 34 cases included for revision, the head–neck adapter system was used specifically in six patients (17.6%) to increase femoral offset, retaining both the acetabular and femoral components. In this subgroup of patients, mean offset decrease after primary THA was 6.6 mm (4.0–9.1), equivalent to a mean 16.3% femoral offset reduction. The median modified Merle d’Aubigné score went from 13.3 preoperatively to 16.2 at one year follow-up.

**Conclusion:**

The use of a head–neck adapter is a safe and reliable procedure that may allow the surgeon to easily correct a slightly diminished femoral offset in a dysfunctional THA without the need to revise well-fixed prosthetic components.

## Introduction

Total hip arthroplasty (THA) is one of the most successful surgeries, with well-reported survival rates. According to national registry reports, a hip replacement is expected to last 25 years in around 58% of patients [1, 2]. However, there are several factors leading to a painful prosthesis that may eventually require revision. The most prevalent causes of revision include infection and inflammatory reactions (20.1%), followed by instability (18.3%), aseptic loosening (15.9%), and mechanical complications (14.9%) [3].

One of the reasons for a dysfunctional hip arthroplasty is a failed restoration of the native femoral offset, which is essential to restore patients’ predisease hip biomechanics and abductor function. In fact, suboptimal restoration of femoral offset results in increased joint reaction forces caused by a reduction in abductor moment arm, which may eventually lead to limping and potential instability [4–7]. Furthermore, decreased femoral offset has been related to poorer functional outcomes [8, 9].

Retaining a well-fixed femoral stem significantly decreases not only the morbidity of the procedure but also blood loss, infection rates, and operating time [9–11]. A head–neck adapter that permits an increase in femoral offset is a useful tool that typically allows the surgeon to easily correct a slightly diminished femoral offset in a dysfunctional THA without the need to revise well-integrated prosthetic components [12].

According to a recent systematic review by Novoa et al [13], the main indication for the use of a head–neck adapter system is isolated acetabular revision (71.6%) followed by prosthetic dislocation (22.2%). The specific use of these adapters with the goal of restoring suboptimal hip offset has not been previously reported. Our aim was to report our experience using a modular head–neck adapter in revision THA and to analyze its usefulness as a tool to specifically increase femoral offset while retaining both the cup and the stem.

## Materials and methods

### Study design

This was an observational retrospective single-center study including all hip revisions performed at our institution from January 2017 to March 2022 that required the use of a modular head–neck adapter system. All the procedures were performed by at least one of the hospital’s joint reconstruction surgeons. Digital templating was carried out in all cases. All preoperative digital radiographs of the pelvis were taken in an anterior–posterior orientation. Since external rotation of the femur usually leads to underestimation of femoral offset [14], patients were positioned supine with both hip joints rotated inward by approximately 10–15°. A dual calibration marker ball system (KingMark^TM^) was routinely used as a reference for determining the individual magnification factor [15]. Digital templating was performed as described by Bono et al. [16, 17], using the TraumaCad^TM^ software (BrainLab, Chicago, IL, USA). A femoral offset was defined as the distance from the center of rotation of the femoral head to a line bisecting the long axis of the femur [18]. All revisions were performed through the same approach used in the primary surgery. Data regarding patient demographics, body mass index (BMI), comorbidities, and indication for revision were recorded. The preoperative and 1-year follow-up modified Merle d’Aubigné [19] hip score was used to evaluate the functional outcome in those cases where the revision was performed solely using the head–neck adapter to specifically increase femoral offset. The modified Merle d’Aubigné score evaluates pain, mobility, and the ability to walk, thus providing a reliable overall assessment of the hip function [20].

### The head–neck adapter

**Fig. 1:**
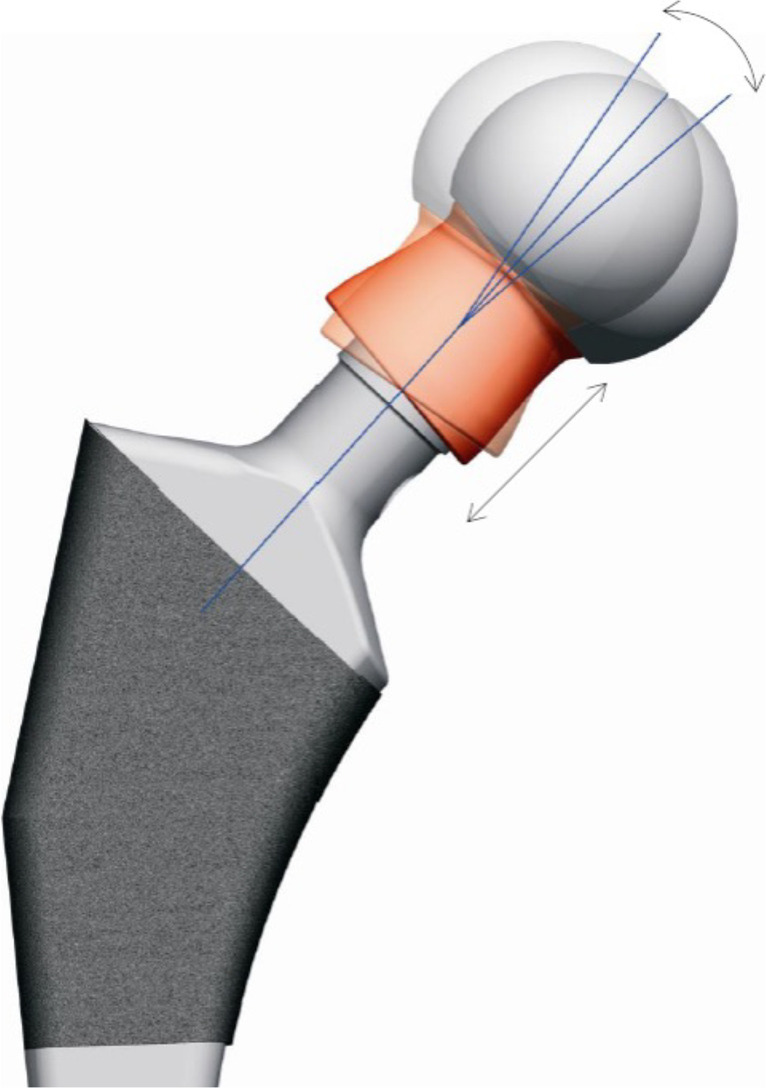
The BioBall^TM^ adapter system (Merete Medical) showing variable neck lengths and offsets

The BioBall^TM^ (Merete Medical, Berlin, Germany) head–neck metal adapter was used in all cases. The BioBall™ is a titanium implant (TiAl6VA4) that comes in different lengths, is versatile enough to adapt to diverse Morse tapers, and is able to add 7.5° offset in any direction (Fig. 1). The adapter comes with modular heads, which are available in different materials and sizes.

### Statistical analysis

Continuous variables were expressed as means or medians and standard deviation (SD) or interquartile range (IQR), whereas qualitative variables were described by absolute frequencies and percentages. Analyses were made and plots were drawn using the SPSS® v. 20.0 statistical package (SPSS, Inc. Chicago, IL, USA).

### Declarations

The study was reviewed by a Research Ethics Committee, which approved it under code HCB/2023/0067, and was conducted in accordance with the 1964 Helsinki Declaration. No funding was sought or secured in relation to this manuscript. At the time of publication, none of the authors disclosed any potential conflicts of interest. None of the authors received any financial support for the research, authorship, and/or publication of this article. The retrospective nature of this study made it unnecessary to obtain the participants’ informed consent. Data was codified, and patients’ anonymity was preserved at all times.

## Results

A total of 34 cases were included for revision. Median patient age was 71.2 years; 61.8% (21 out of 34) of the sample were male. The most frequent indication for surgery was revision of the acetabular cup (*n* = 26, 76.5%) followed by head replacement (while retaining both cup and stem) in order to achieve a higher offset (*n* = 6, 17.6%). There were two cases (5.9%) where the head–neck adapter was used during primary THA implantation. The adapter was not used in any case where all the prosthetic components had to be revised. An anterolateral Hardinge approach was used in 76.6% of cases (*n* = 26). As regards the implants’ characteristics, the most used neck length was 2XL (+ 10.5 mm) (30.3% of cases (*n* = 10)), followed by L (+ 3 mm) and 3XL (+ 14 mm) (27.3% (*n* = 9) and 18.2% (*n* = 6)), respectively. Regarding the taper, a 12/14 taper was used in 88.2% of cases (*n* = 30). The most frequently used head sizes were 36 mm (47.1% of patients (*n* = 16)) and 28 mm (44.1% of patients (*n* = 15)). The 7.5° offset adapter was used in 76.5% (*n* = 26) of patients. The head material was ceramics in 50% (*n* = 17) of cases, and dual-mobility heads were used in 41.2% of patients (*n* = 14). The characteristics of the patient sample are shown in Table 1.

**Table 1:** Main demographic data of the 34 patients in which the Bioball^TM^ system was used for any reason during the study period

*n*	Age	Sex	Side	BMI	Surgical approach	Head diameter (mm)	Taper	Type of surgery	Adapter offset
1	64	M	R	27.8	AL	36	N/A	Cup revision	0
2	79	M	R	21.3	AL	36	12/14	Cup revision	0
3	83	M	R	22.0	AL	36	10/12	Cup revision	0
4	81	M	R	28.5	AL	28	N/A	Cup revision	0
5	76	M	L	28.3	AL	36	12/14	Cup revision	7.5°
6	75	M	R	24.2	AL	28	10/12	Cup revision	0
7	68	M	L	27.1	AL	28	12/14	Cup revision	7.5°
8	71	F	R	34.2	PL	32	10/12	Cup revision	0
9	81	M	L	29.4	AL	36	12/14	Cup revision	7.5°
10	37	F	L	26.6	AL	36	N/A	Primary THA	0
11	32	F	R	30.9	AL	28	12/14	Cup revision	7.5°
12	85	M	L	29.1	DA	36	12/14	Primary THA	7.5°
13	81	M	L	23.8	PL	36	12/14	Cup revision	7.5°
14	75	F	R	30.9	AL	28	12/14	Cup revision	7.5°
15	76	M	L	29.8	AL	36	12/14	Cup revision	7.5°
16	68	M	L	35.2	AL	36	12/14	Head–neck adapter to increase offset	7.5°
17	52	F	L	21.9	AL	36	12/14	Head–neck adapter to increase offset	7.5°
18	69	M	R	33.3	AL	36	12/14	Cup revision	7.5°
19	74	M	R	29.4	AL	36	12/14	Head–neck adapter to increase offset	7.5°
20	60	F	L	24.0	AL	32	12/14	Cup revision	7.5°
21	63	M	L	23.0	AL	36	N/A	Cup revision	7.5°
22	41	F	L	35.0	AL	36	12/14	Cup revision	7.5°
23	89	F	L	23.1	PL	28	12/14	Cup revision	7.5°
24	78	F	L	22.8	AL	28	12/14	Cup revision	0
25	80	F	R	24.2	PL	28	12/14	Cup revision	7.5°
26	82	M	L	34.6	AL	28	12/14	Cup revision	7.5°
27	73	M	L	29.1	AL	36	12/14	Head–neck adapter to increase offset	7.5°
28	73	F	R	26.6	AL	28	12/14	Cup revision	7.5°
29	84	F	R	24.4	AL	28	12/14	Cup revision	7.5°
30	58	M	R	24.6	PL	28	12/14	Cup revision	7.5°
31	86	M	R	22.4	PL	28	12/14	Cup revision	7.5°
32	73	F	R	28.3	WJ	28	12/14	Cup revision	7.5°
33	83	M	L	27.0	AL	28	12/14	Head–neck adapter to increase offset	7.5°
34	71	M	R	25.6	AL	36	12/14	Head–neck adapter to increase offset	7.5°

*BMI* body mass index, *M* male, *F* female, *L* left, *R* right, *AL* anterolateral, *DA* direct anterior, *PL* posterolateral, *WJ* Watson–Jones, *THA* total hip arthroplasty, *N/A* not available

**Fig. 2:**
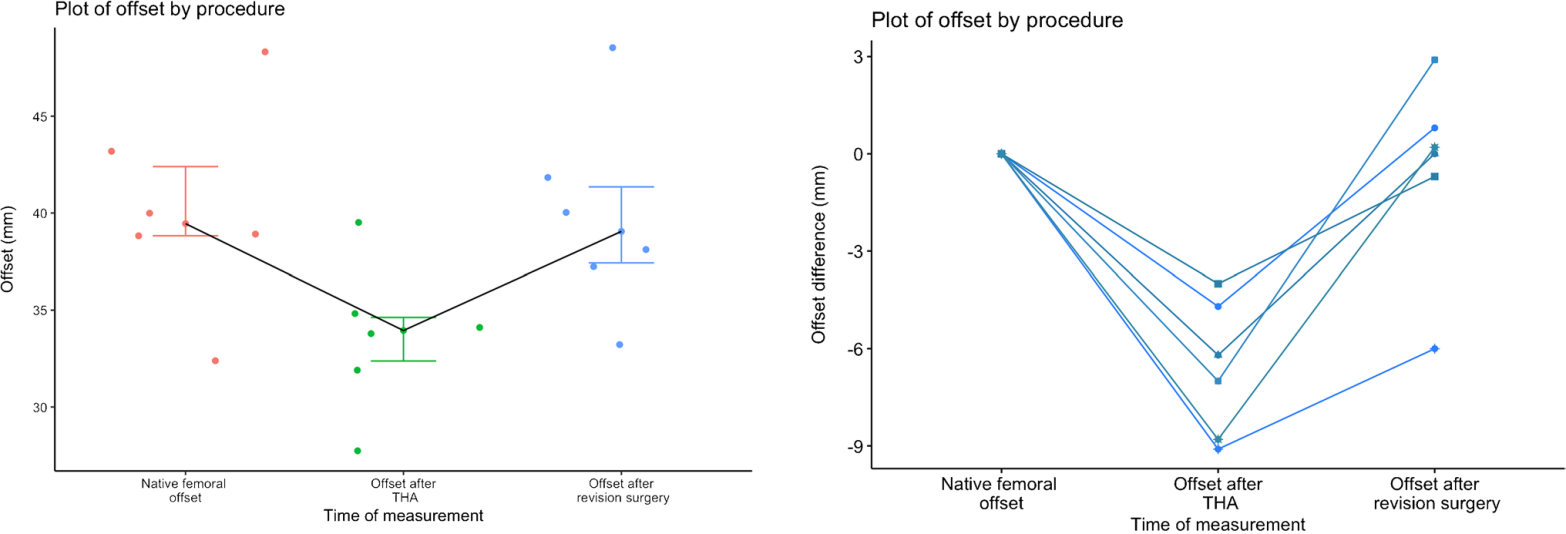
Left: box plot displaying the initial femoral offset decrease after a potentially dysfunctional total hip arthroplasty and the subsequent femoral offset restoration following revision using the Bioball^TM^ 7.5-degree offset head–neck adapter. Right: line plot depicting the femoral offset variation of each patient between the procedures. THA: total hip arthroplasty

**Fig. 3:**
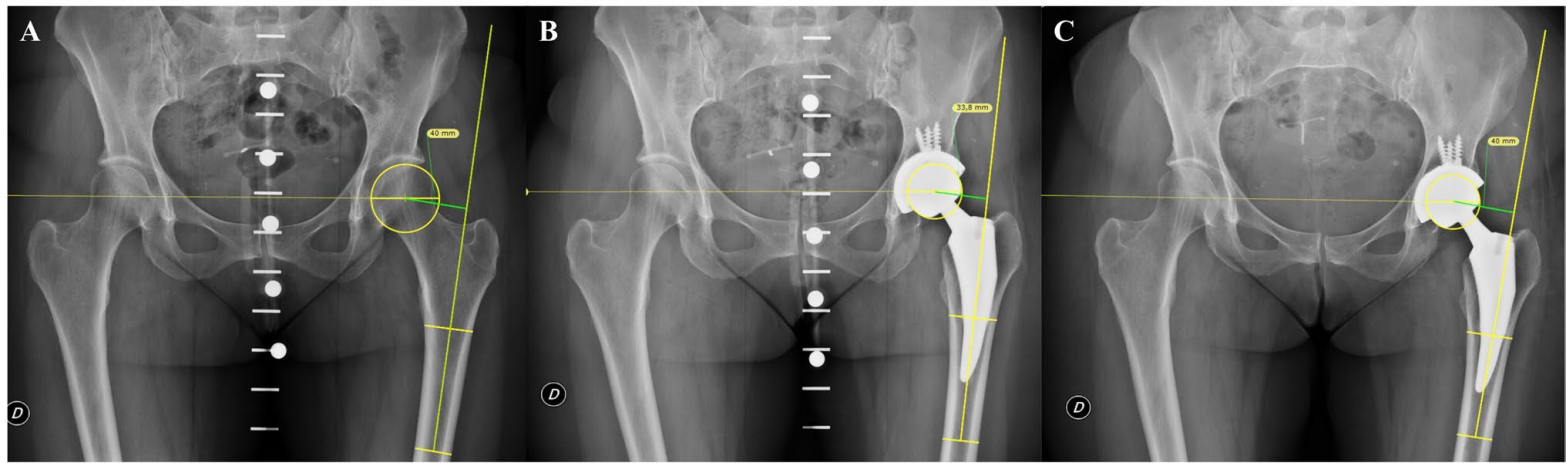
Anteroposterior pelvis view of a patient where the native hip offset (**A**) diminished after implantation of a total hip arthroplasty (**B**) and was eventually restored by means of a Bioball^TM^ 7.5-degree offset head-neck adapter (**C**)

As mentioned earlier, the head–neck adapter was used in six patients (17.6%) specifically to increase femoral offset. Of these six patients, five presented with hip pain that improved after prosthetic revision. A total of five patients presented with walking difficulties, which resolved completely in three cases. There was one patient that reported no improvement following surgery and one patient with multiple sclerosis that progressively worsened in terms of function even though a certain degree of pain relief was achieved. All six patients were operated through an anterolateral Hardinge approach. A partial gluteus medius tear was found in all cases, which was repaired with direct transosseous suture. The median offset decrease after primary THA had been 6.6 mm (4.0–9.1), causing a median femoral offset reduction of 16.3% (Figs. 2 and 3). The median femoral Merle d’Aubigné score rose from 13.3 preoperatively to 16.2 at 1-year follow-up. The detailed characteristics of these six patients are shown in Table 2.

**Table 2:** Main characteristics of the six patients where implantation of a Bioball^TM^ system was specifically performed to restore a diminished femoral offset. The Merle d‘Aubigné score provides an assessment of hip function. The score is considered excellent if equal to 18, very good if equal to 17, good if between 15 and 16, average if between 13 and 14, poor if between 9 and 12, and very poor if < 9

*n*	Sex	Age	BMI	Indication for THA	Comorbidities	Hip approach	Stem offset	Taper	Head Diameter (mm)	Offset of the native hip	Offset after THA (offset decrease in mm, % decrease)	Offset after revision with Bioball^TM^	Merle d’Aubigné after THA (pain–mobility–gait (overall))	Merle d’Aubigné after revision with Bioball^TM^ (pain–mobility–gait (overall))
1	M	65	34.8	Perthes	Obesity, lumbar arthropathy	AL	STD	12/14	36	32.4	27.7 (4.7, 14.5%)	33.2	4–6–5 (15)	6–6–6 (18)
2	F	52	19.7	Dysplasia	None	AL	STD	12/14	36	40.0	33.8 (6.2, 15.5%)	40.0	3–6–3 (12)	6–6–6 (18)
3	M	72	29.4	OA	Multiple sclerosis	AL	STD	12/14	36	38.9	31.9 (7.0, 18.0%)	41.8	3–4–1 (8)	5–4–0 (9)
4	M	73	28.7	OA	OSAS	AL	STD	12/14	36	43.2	34.1 (9.1, 21.1%)	37.2	4–6–6 (16)	6–6–6 (18)
5	M	83	26.9	Femoral neck fracture	Stroke, lumbar arthrodesis	AL	STD	12/14	32	38.8	34.8 (4.0, 10.3%)	38.1	6–6–4 (16)	6–6–4 (16)
6	M	70	25.6	OA	Diabetes mellitus	AL	HO	12/14	36	48.3	39.5 (8.8, 18.2%)	48.5	3–6–4 (13)	6–6–6 (18)

*M* male, *F* femalem, *AL* anterolateral, *OA* osteoarthritis, *OSAS* obstructive sleep apnea syndrome, *THA* total hip arthroplasty, *STD* standard, *HO* high offset

## Discussion

An accurate restoration of native femoral offset is often regarded as an essential requirement to obtain an optimal prosthetic joint. However, a certain amount of controversy still persists among surgeons. It is well known that patients with decreased offset present with poorer joint function at one-year follow-up [9]. Even though a 15% or > 5-mm decrease in offset has been related to hip abductor weakness [21, 22], its association with lower functional scores or the use of a walking aid [9, 23] remains unclear. In our series, the six cases specifically reoperated due to decreased offset experienced a mean femoral offset reduction of 6.6 mm (mean decrease of 16.3%) and they all presented with abductor weakness. Bullen et al. [24] found that an offset decrease greater than 20 mm typically resulted in worse pain and lower motion scores. Five of the six cases in the subgroup specifically operated due to decreased offset obtained significantly improved Merlé d’Aubigné scores following revision with a head–neck adapter.

The head–neck adapter has become a widely used tool, mainly in partial hip revisions where the cup is replaced and the adapter is used to restore the taper surface on a previously damaged cone. During the procedure, the existing taper is replaced by the adapter’s own taper, hence allowing retention of the primary stem. Moreover, the stability resulting from addition of a 7.5° offset may be used to address recurrent dislocation [25–27]. For this reason, as reported by Kock et al. [28], the adapter may be also used in primary THA in cases with a high risk of dislocation. In our series, the head–neck adapter was used in primary THA in two cases: in one case (case 10), the adapter was used to solely increase femoral neck length, whereas in the other case (case 12), the adapter added a 7.5° offset, which provided a more accurate centre of rotation.

It has been reported that patients operated due to hip dysplasia are at a high risk of experiencing an excessively increased offset after THA. Indeed, dysplasia is typically accompanied by decreased femoral offset, which is usually excessively augmented when regular necks are used [9, 29]. These patients constitute a significant challenge in terms of offset restoration, particularly in the presence of a short varus and an anteverted neck. Two of the six patients analyzed presented with a paediatric hip abnormality and in five of them a standard offset neck was used in the primary THA, which was eventually replaced by an offset head-neck adapter. In Cassidy et al. [9], 22.6% of the patients in the decreased offset group still exhibited a decreased offset postoperatively despite the use of an extended offset stem. This possibility should be carefully considered, as one of the main objectives of any reconstruction procedure is to normalize femoral offset irrespective of the initial diagnosis.

To the best of our knowledge, this is the first study to report on the use of the Bioball^TM^ head–neck adapter to specifically correct a slightly diminished and hence suboptimal femoral offset. However, the present study is subject to some inherent limitations. Most importantly, because of its retrospective nature, certain biases may have influenced the results. The study’s retrospective design may have resulted in an inadequate control over confounding variables and limited the amount of information obtained. Secondly, it is impossible to ascertain whether the outcome of the procedure can be strictly attributed to the revision of the investigated component or whether it was also related to the way the periarticular soft tissues were managed. Given that all six cases presented with gluteus medius tears of various sizes, the authors acknowledge that the benefit might have been at least in part related to the direct tendon repair performed. Finally, the limited number of cases inherent in a single-center study on a low-prevalence condition requires the performance of further research to corroborate the findings obtained. All these limitations may affect the extent to which the results presented here can be generalized beyond the specific cases studied.

In conclusion, a head–neck adapter is a safe and reliable tool that may allow the surgeon to easily correct a slightly diminished offset in a dysfunctional THA without the need to revise well-integrated prosthetic components. The use of the component might be considered whenever a revision of a decreased-offset arthroplasty is performed.
